# Decoupling of DNA damage response signaling from DNA damages underlies temozolomide resistance in glioblastoma cells^☆^

**DOI:** 10.1016/S1674-8301(10)60057-7

**Published:** 2010-11

**Authors:** Bo Cui, Stewart P. Johnson, Nancy Bullock, Francis Ali-Osman, Darell D. Bigner, Henry S. Friedman

**Affiliations:** a Departments of Surgery; b Pathology, Duke University Medical Center, Durham, NC 22710, USA

**Keywords:** glioblastomas multiforme, temozolomide, DNA damage response, resistance

## Abstract

Glioblastoma multiforme (GBM) is the most aggressive primary brain tumor in adults. Current therapy includes surgery, radiation and chemotherapy with temozolomide (TMZ). Major determinants of clinical response to TMZ include methylation status of the O6-methylguanine-DNA methyltransferase (MGMT) promoter and mismatch repair (MMR) status. Though the MGMT promoter is methylated in 45% of cases, for the first nine months of follow-up, TMZ does not change survival outcome. Furthermore, MMR deficiency makes little contribution to clinical resistance, suggesting that there exist unrecognized mechanisms of resistance. We generated paired GBM cell lines whose resistance was attributed to neither MGMT nor MMR. We show that, responding to TMZ, these cells exhibit a decoupling of DNA damage response (DDR) from ongoing DNA damages. They display methylation-resistant synthesis in which ongoing DNA synthesis is not inhibited. They are also defective in the activation of the S and G2 phase checkpoint. DDR proteins ATM, Chk2, MDC1, NBS1 and gammaH2AX also fail to form discrete foci. These results demonstrate that failure of DDR may play an active role in chemoresistance to TMZ. DNA damages by TMZ are repaired by MMR proteins in a futile, reiterative process, which activates DDR signaling network that ultimately leads to the onset of cell death. GBM cells may survive genetic insults in the absence of DDR. We anticipate that our findings will lead to more studies that seek to further define the role of DDR in ultimately determining the fate of a tumor cell in response to TMZ and other DNA methylators.

## INTRODUCTION

Glioblastoma multiforme (GBM) is the most common, aggressive and difficult to treat primary brain tumour in adults with the majority of patients surving less than two years[1]. Current therapy includes surgery, radiation and adjuvant chemotherapy[2]. Temozolomide (TMZ), an *S_N_1* type monofunctional DNA methylating agent., increases the survival of GBM patients in concurrent therapy with radiation[3]. However, resistance to TMZ emerges with prolonged treatment and posts a major therapeutic challenge. One of the major determinants of clinical response to TMZ is the methylation status of the promoter region of *O^6^*-methylguanine (*O^6^*-meG) DNA methyltransferase (MGMT). The hypermethylation of MGMT promoter is seen in approximately 40% to 60% of patients and correlates with a favorable response to TMZ[4]–[6]. The other known determinant of clinical response to TMZ is mismatch repair (MMR) status. Recurrence of GBM was associated with loss of MMR proteins in patients undergoing prolonged TMZ therapy[7],[8].

Though MGMT promoter is methylated in up to 60% of GBM cases, for the first nine months of follow-up, addition of TMZ to radiotherapy does not change survival outcome[4], suggesting that MGMT methylation is the not the sole factor determining outcome. Furthermore, though MMR-proficient cells are approximately 100 fold more sensitive to S_N_1 type methylators than their MMR-deficient counterparts[9], MMR deficiency makes a minor contribution to clinical resistance in GBM patients[10], suggesting that there exist unrecognized mechanisms of resistance.

*O^6^*-meG accounts for approximately 9% of the DNA alkylation adducts by TMZ[11], but it provides the major signal for eliciting DNA damage response (DDR)[12],[13] and is also the major initiator of cellular apoptotic response[14]–[16]. MGMT specifically demethylates *O^6^*-meG by transferring the methyl group to an active cysteine residue in itself. *O^6^*-meG adducts, if unrepaired, cause *O^6^*-meG:C to *O^6^*-meG:T transition mutations after DNA replication[17]. The MMR complexes MutSα and MutLα have an affinity for *O^6^*-meG:T mismatches[18]. These mispairs are initially detected and processed by MSHα and MLHα, which then signal to activate the ataxia-telangiectasia-mutated kinase (ATM)-checkpoint 2 kinase (Chk2) and the Rad3-related kinase (ATR)-checkpoint 1 kinase (Chk1) pathway[12],[13], leading to the activation of a transient checkpoint in the second S phase and an eventual cell cycle arrest at the following G2 phase[9].

The MRN complex, which includes Mre11, Rad50 and Nbs1, is also involved in DDR to TMZ. Mre11 forms discrete subnuclear foci in response to TMZ. Phosphorylated histone H2A variant protein H2AX (γ-H2AX), a reliable marker of DNA double-strand breaks (DSBs) and early apoptosis, also forms distinct foci in response to TMZ[19]. The DNA damage mediator protein MDC1 transduces DNA damage signals and controls the activation of intra-S phase checkpoint. It forms a complex with γ-H2AX and offers a platform to support retention of DDR proteins like ATM and Nbs1 at sites of DNA damages[20]. The involvement of MDC1 in DDR to temozolomide, however, remains unelucidated.

The functional availability of the DDR signaling network that responds to DNA damage likely directs how GBM responds to TMZ and decides the therapeutic outcome. We hypothesize that DDR signaling may be impaired in TMZ-resistant GBM cells, which could contribute to their phenotypic resistance. We have generated paired TMZ-resistant GBM cell lines whose resistance is due to neither high MGMT activities nor deficient MMR status. We report here that these resistant GBM cell lines exhibit methylation-resistant DNA synthesis (MDS), defective cell cycle checkpoints, and impaired DDR signalling, suggesting that, in the TMZ-resistant GBM cell lines, DDR signaling is decoupled from ongoing DNA alkylation damages by TMZ, which may underlie the phenotypic resistance in the TMZ-resistant GBM cells.

## MATERIALS AND METHODS

### Drug and drug treatments

TMZ (Schering-Plough, Kenilworth, NJ) and the MGMT inactivator *O^6^*-benzylguanine (*O^6^*-BG) (National Cancer Institute, USA) were freshly prepared in dimethyl sulfoxide before use. For drug treatments, logarithmically growing cells were used and incubated with TMZ at a concentration equal to IC_90_ for D54 and U251 for the time points indicated. Where indicated, U251 and U251 (OTR) were initially exposed to 100 µmol/L *O^6^*-BG, treated with TMZ for 4 h and plated in medium with 20 µmol/L *O^6^*-BG. For clonogenic assays, TMZ from 12.5 µmol/L up to 1 mmol/L was used.

### Cell lines, generation of drug-resistant cells and clonogenic assay

The GBM cell lines D54, D54 (OTR), U251, U251 (TR) and U251 (OTR) were maintained in Improved MEM Zinc Option (Richter's modification) (Invitrogen, USA) supplemented with 20% fetal bovine serum (FBS) (Invitrogen). For generation of drug-resistant TR cell lines, we began treating cells with 10 µmol/L TMZ for 4 h. The cells were maintained at this drug concentration until they showed markedly increased survival to treatment. After every three passages, the drug concentration was increased by 5 to 10 µmol/L. The OTR cell lines were generated the same way, except that: (1) the cells were exposed to 100 µmol/L *O^6^*-BG for 10 min before TMZ treatment; (2) cells were maintained in medium with 20 µM *O^6^*-BG. The cytotoxicity of TMZ against the GBM cells was determined by clonogenic assays as previously described[21].

### Measurement of MGMT activity and mismatch repair assays

The activity of MGMT was measured as described previously[22] and the enzyme activity was defined as the fmol of *O^6^*-[^3^H] meG removed from ^3^H-methylated DNA per mg of extracted protein. Mismatch correction in U251 and U251 (OTR) or D54 and D54 (OTR) nuclear extracts was determined using heteroduplex DNA containing a GT mismatch as previously described[23].

### [^3^H]-thymidine ([^3^H] TdR) incorporation assay

GBM cells were pre-labeled for 24 h with 0.05µCi [^14^C] TdR/mL (Perkin-Elmer, Wellesley, MA). [^14^C] TdR incorporation was stopped by washing the cells twice with cold PBS (Sigma). The cells were then pulse-labeled for 2 h with 2.0 µCi [^3^H] TdR/mL (Perkin-Elmer) and, after [^3^H] TdR incorporation was stopped by washing the cells twice with cold PBS, the cells were incubated for 30 min with unlabeled medium to chase the labeled precursor pool. Thereafter, the cells were lysed with 0.25 mol/L NaOH and samples were counted by dual-label liquid scintillation, and the amount of [^3^H] TdR was normalized to the amount of [^14^C] TdR in each sample. DNA synthesis was measured as a ratio of [^3^H] to [^14^C] in the treated samples divided by the ratio of [^3^H] to [^14^C] in the untreated control samples.

### Comet assays

The alkaline and neutral comet assays were carried out according to the manufacturer's recommendations (Trevigen, Gaithersburg, MD). Gel electrophoresis was carried out in alkaline electrophoresis solution (pH >13) at 1 V/cm for 10 min or in 1×Tris-borate-EDTA buffer at 4.5 V/cm for 5 min at room temperature. Slides were stained with SYBER Green and analyzed by fluorescence microscopy (Leica DMS 4000B). The percentage of DNA in the comet tail or tail length was determined using the software package “Comet Assay II” (Perceptive Instruments, UK). A minimum of 50 cells per experiment was analyzed. All the experiments were done in triplicate.

### Antibodies, immunoblotting analysis and immunofluorescence microscopy

The antibodies for the following proteins were used for immunoblotting studies: phospho-Chk1 (serine 345) and phospho-Chk2 (threonine 68) (Cell Signaling Technology, USA), phospho-ATM (serine 1981) (Rockland, USA) and α-tubulin (Santa Cruz Biotechnology, USA). The following antibodies were used for immunofluorescence microscopy: mouse anti-phospho-ATM antibody (serine 1981), rabbit anti-phospho-Nbs1 antibody (serine 343), rabbit anti-MDC1 antibody, mouse and rabbit anti-γ-H2AX antibody (serine 139; Abcam, USA), and rabbit anti-phospho-Chk2 antibody (threonine 68; Santa Cruz Biotechnology). Secondary antibodies Alex Fluor 488 goat anti-rabbit IgG and 594 goat anti-mouse IgG (Invitrogen) were used. Immunoblotting and immunofluorescence microscopy were done as previously described[24],[25]. Nuclear staining was done using 4′, 6-diamidino-2-phenylindole (DAPI) (Vector Laboratories, USA).

### Cell-cycle analysis

Treated GBM cells were washed once with PBS, trypsinized, and washed again in PBS with 2% FBS and fixed in ice-cold ethanol for at least 1 h at -20°C, washed, and stained with propidium iodide (30 µg/mL) and treated with RNase (0.6 mg/mL) in PBS plus 0.5% (v/v) Tween 20 and 2% FBS. Stained cells were analyzed on a FACSCalibur flow cytometer (BD Bioscience, USA) using Cellquest software, and the ModFit program (Verity Software House Inc., USA) was used to analyze the cell-cycle profiles.

### Statistical analysis

Data were expressed as mean±SD and analyzed using the SPSS10.0 sofeware. *P* < 0.05 was considered statistically significant.

## RESULTS

### The resistant GBM cell lines exhibited novel mechanisms of chemoresistance

**Fig. 1:**
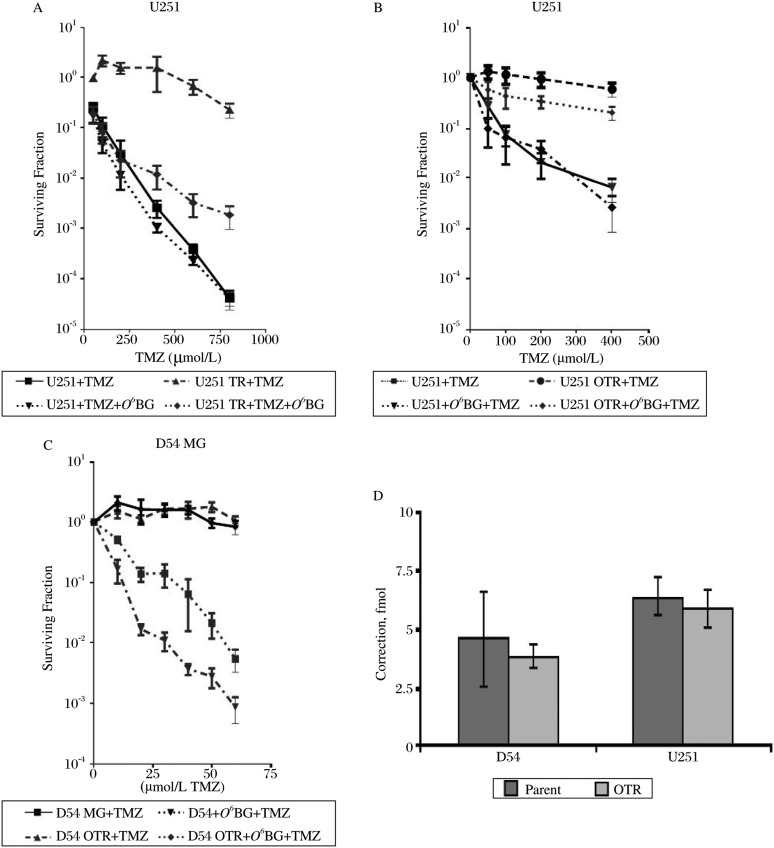
The temozolomide (TMZ)-resistant GBM cell lines exhibit unknown mechanisms of resistance. A: U251 (TR) cells exhibit enhanced survival to TMZ compared with U251 cells. The MGMT inactivator, O6-BG, sensitizes U251 (TR) cells to TMZ with a survival similar to U251 cells. B and C: U251 (OTR) and D54 (OTR) exhibit enhanced survival against TMZ compared with U251 and D54, respectively. The MGMT inactivator, O6-BG, does not sensitize any of these cell lines to TMZ. C: U251 and U251 (OTR), and D54 and D54 (OTR) exhibit similar MMR capacity. D: Each data point represents the average of three independent experiments; bars, mean±SD.

We treated U251 and U251 (TR) cells with TMZ and clonogenic assays indicated that, compared with U251 cells, U251 (TR) cells were markedly resistant to TMZ (***Fig. 1A***). With the addition of 25 µmol/L *O^6^*-BG, U251 (TR) cells showed a clonogenic survival profile similar to that of U251 cells, suggesting that resistance in U251 (TR) cells to TMZ was due to high MGMT activities.TMZ markedly inhibited the growth of U251 with an IC_50_ and IC_90_ of 50 and 100 µmol/L, respectively, whereas TMZ exerted no noticeable growth-inhibitory effect on the TMZ-resistant U251 (OTR) (***Fig. 1B***). D54 was sensitive to growth inhibition by TMZ with an IC_50_ and IC_90_ of 20 and 40 µmol/L, respectively, while D54 (OTR) was resistant to the growth-inhibitory effects of the drug (***Fig. 1C***). Additionally, 20 µmol/L *O^6^*-BG failed to sensitize U251 (OTR) or D54 (OTR) to TMZ.

Furthermore, U251 exhibited virtually no MGMT activity (<10 fmol/mg protein) and U251 (OTR) expressed moderate amounts of MGMT activities (419±37 fmol/mg protein) while TMZ-resistant U251 (TR) expressed high levels of MGMT activity (715±70 fmol/mg protein). Co-administration of 20 µmol/L *O^6^*-BG with TMZ reduced the MGMT activities essentially to zero in U251 (OTR) and U251 (TR). D54 and D54 (OTR) also expressed virtually no MGMT activity (<10 fmol/mg protein). *In vitro* MMR assays using nuclear extracts from these GBM cell lines and DNA substrates containing a G:T mismatch[23] indicated that there was also no difference in MMR capacity between D54 and D54 (OTR) or between U251 and U251 (OTR) (***Fig. 1D***).

### The resistant GBM cells exhibited MDS in response to TMZ

**Fig. 2:**
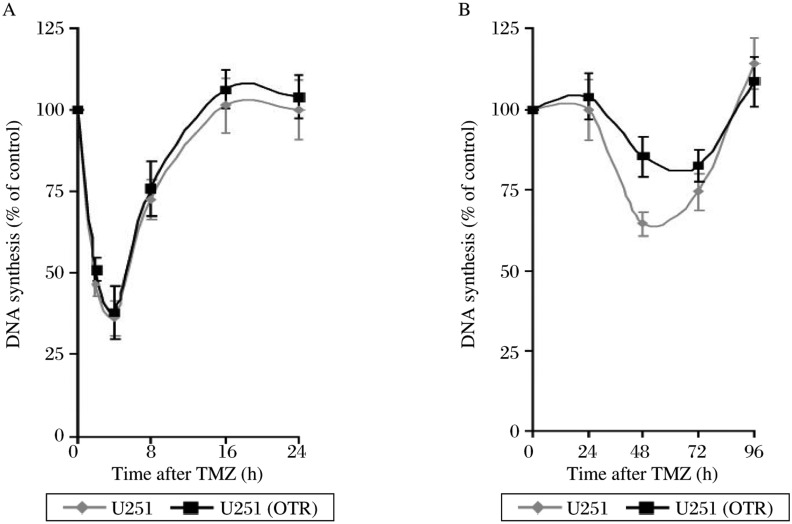
TMZ causes a biphasic inhibition of DNA synthesis. TMZ causes an acute decline in DNA synthesis followed by a second prolonged phase of inhibition of DNA synthesis. U251 (OTR) (A) and D54 (OTR) (B) exhibit methylation-resistant DNA synthesis in this second phase. Each data point represents the average of three independent experiments; bars: mean±SD. TMZ: temozolomide

DNA alkylation damages were shown to inhibit DNA synthesis[26],[27]. We analyzed DNA synthesis in U251 and U251 (OTR) by the [^3^H] TdR incorporation method, which revealed that these cells responded acutely to TMZ by inhibiting the overall rate of DNA replication within minutes after drug exposure, producing a rapid and steep decline in DNA synthesis (***Fig. 2A***). This sharp depression was followed by a dramatic recovery from the nadir of DNA synthesis. Thereafter, these cells underwent a second prolonged decline in DNA synthesis, which started at 36 h and recovered to control levels by 96 h after treatment. During this period, TMZ caused a greater inhibition of DNA synthesis in U251 than U251 (OTR) (64.51±3.64% of controls *vs* 85.35±6.25% of controls, 48 h post treatment), suggesting that U251 (OTR) exhibited MDS in response to TMZ.

Similar findings were also observed in D54 and D54 (OTR) (***Fig. 2B***). A marked inhibition of DNA synthesis was noticed in D54 48 h post treatment (81.2±0.48% of controls). D54 (OTR), on the other hand, exhibited a similar rate of DNA synthesis to that in control cells (119.84±8.81% of controls), indicating ongoing DNA synthesis in these cells despite the presence of DNA damages.

### O^6^-meG contributed to the inhibition of DNA synthesis in GBM cells

As TMZ generates several different methyl adducts like *N^7^*-methylguanine, *N^3^*-methylguanine and *O^6^*-meG, we investigated whether *O^6^*-meG contributed to TMZ-induced inhibition of DNA synthesis. Analysis of [^3^H] TdR incorporation in U251 and U251 (TR) revealed a marked inhibition of DNA synthesis in U251 (40.74±14.11% of controls) while DNA synthesis remained virtually unchanged in U251 (TR) (94.13±7.12% of controls) at 4 h after treatment, suggesting that *O^6^*-meG contributed to the acute inhibition of DNA synthesis. At 48 h after treatment, DNA synthesis remained virtually unchanged in U251 (TR) (106.79±7.63% of controls) while that in U251 stood at 67.94±7.47% of controls, suggesting that *O^6^*-meG also contributed to the second prolonged phase of DNA synthesis inhibition.

### The resistant GBM cells incurred extents of DNA methylation damages similar to their parental counterparts

**Fig. 3:**
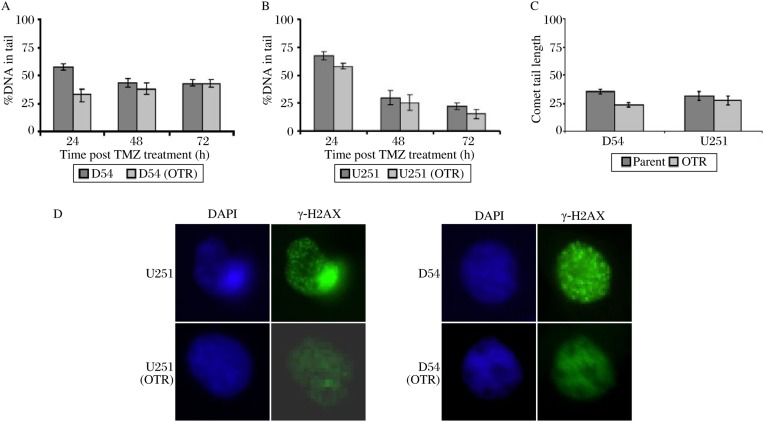
DNA methylation damages persist in the GBM cells, A and B: The presence of DNA methylation damage at sequential time points after TMZ treatment was measured by single cell alkaline gel electrophoresis. TMZ induces DNA DSBs in the GBM cells. C: The presence of DNA DSBs at 72 h after TMZ treatment was measured by single cell neutral gel electrophoresis and D by indirect immunofluorescence microscopy using antibody against γ-H2AX. γ-H2AX failed to form foci in the resistant GBM cells after TMZ treatment. Each data point in A, B and C represents the average of three independent experiments; bars: mean±SD. TMZ: temozolomide.

With the exclusion of high MGMT activities and differential MMR capacity as the mechanisms of resistance to TMZ in U251 (OTR) and D54 (OTR), we investigated whether the enhanced clonogenic survival in the resistant GBM cells was attributed to enhanced repair of DNA methyl adducts. We monitored the formation and resolution of TMZ-induced DNA adducts in the GBM cells over time by using the alkaline comet assay, which detects single-strand breaks and alkali-labile sites. We treated U251 and U251 (OTR) with 100 µM TMZ in the presence of *O^6^*-BG and D54 and D54 (OTR) with 40 µM TMZ. The results indicated that U251 and U251 (OTR) all incurred the greatest amount of DNA damages at 24 h post treatment [*P* < 0.05, U251 *vs* U251 (OTR)], which, though at reduced levels, persisted well into 72 h post treatment (***Fig. 3A*** and ***3B***). The extent of DNA damages as reflected in the percentage of DNA in the comet tail was not significantly different in U251 and U251 (OTR) at all time points examined (*P* > 0.05). Though D54 incurred DNA damages to a greater extent than D54 (OTR) at 24 h after treatment [*P* < 0.05, U251 *vs* U251 (OTR)], both D54 and D54 (OTR) exhibited similar extent of DNA damages at later time points (***Fig. 3A***), suggesting that TMZ-induced DNA methylation damages were not preferentially repaired in the resistant GBM cells.

### γ-H2AX failed to localize to sites of DNA damages in the resistant GBM cells

DNA DSBs arise from processing of *O^6^*-meG:C and *O^6^*-meG:T mismatches by the DNA repair apparatus[14],[28]. We carried out neutral comet assays to detect the presence of DNA DSBs in the GBM cells 72 h following treatment. Similar amounts of DNA DSBs were found in U251 and U251 (OTR) while DNA DSBs were present to a greater extent in D54 than D54 (OTR) (***Fig. 3C***). The presence of DNA DSBs was confirmed by immunofluorescence microscopy using anti-γ-H2AX antibody. γ-H2AX formed distinct foci in response to TMZ in U251 and D54 (***Fig. 3D***). However, γ-H2AX failed to localize to sites of DNA damages in the resistant GBM cells despite the presence of DNA DSBs as revealed by the neutral comet assay.

### The resistant GBM cells exhibited impaired DDR to TMZ

**Fig. 4:**
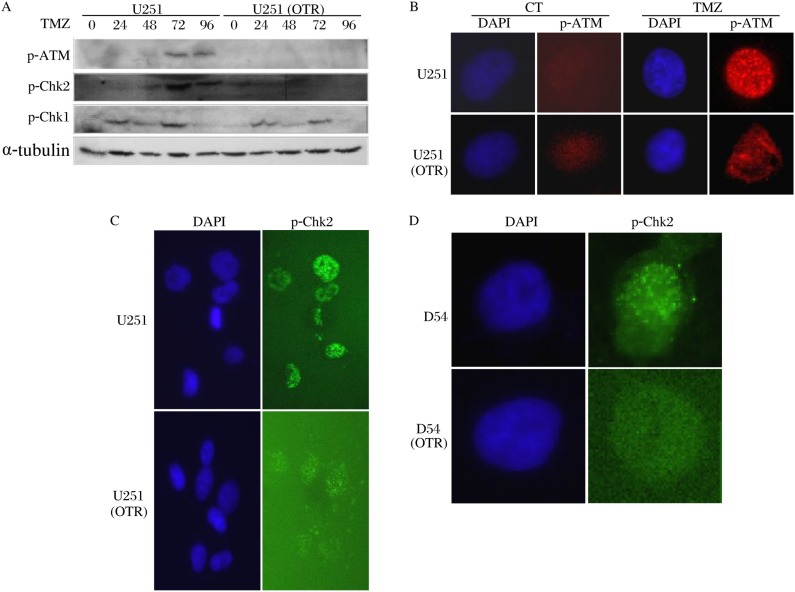
The resistant GBM cells exhibit defective signaling through the ATM-Chk2 pathway. A: U251 (OTR) fails to activate the ATM-Chk2 pathway in response to TMZ. The Western blotting were probed with phospho-specific antibodies against ATM (p-ATM), Chk1 (p-Chk1) and Chk2 (p-Chk2). B: TMZ induces focus formation by p-ATM in U251, but not in U251 (OTR). C and D, TMZ induces focus formation by p-Chk2 in D54 and U251, but not in their TMZ-resistant counterparts. The cells in B, C and D were analyzed at 72 h post TMZ treatment. CT: control; TMZ: temozolomide.

*O^6^*-MeG adducts signal to activate the ATR-Chk1 and the ATM-Chk2 pathway[12],[13]. We examined the activation of the two pathways by immunoblotting analysis using phospho-specific antibodies against ATM, Chk1 and Chk2. We found that the ATR-Chk1 pathway was activated from 24 to 72 h post TMZ exposure in both U251 and U251 (OTR) (***Fig. 4A***). However, the ATR-Chk2 pathway was activated from 72 to 96 h post treatment only in U251, suggesting that DNA damages in U251 (OTR) failed to signal through the ATM-Chk2 pathway. Examination by indirect immunofluorescence microscopy further revealed that phosphorylated ATM formed discrete foci in U251, but not in U251 (OTR) (***Fig. 4B***). Phosphorylated Chk2 also formed distinct foci in U251 and D54, but not in their resistant counterparts (***Fig. 4C*** and ***Fig. 4D***).

**Fig. 5:**
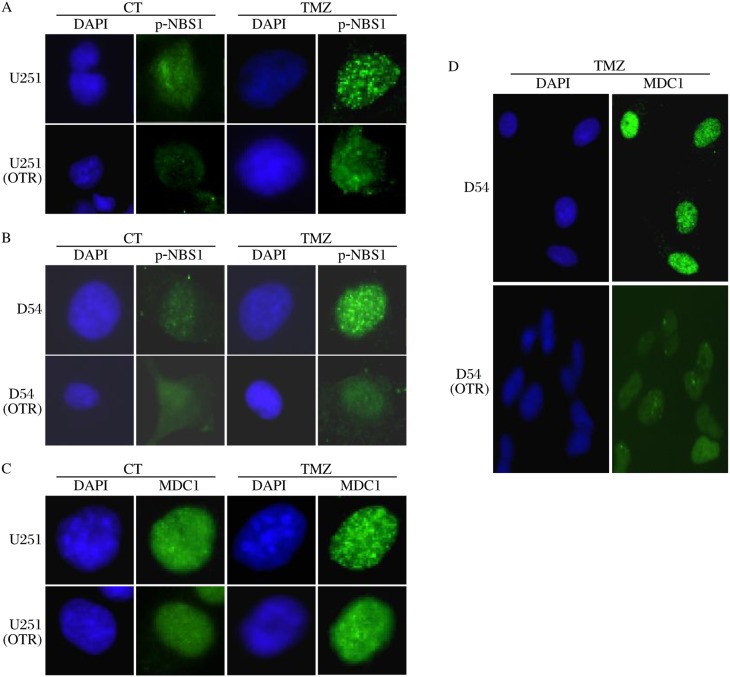
The resistant GBM cells show defective DDR. Phosphorylated NBS1 (p-NBS1) (A and B) and MDC1 (C and D) form distinct foci 72 h post treatment in D54 and U251, but not in their TMZ-resistant counterparts. CT: control, TMZ: temolomide.

In addition, phosphorylated Nbs1 and the DNA damage mediator protein MDC1 formed discrete foci in U251 and D54, but failed to do so in the resistant GBM cells (***Fig. 5A*** to ***Fig. 5D***). These findings suggest that these DDR proteins were activated in response to TMZ in D54 and U251. However, they failed to respond to DNA damages induced by TMZ in the resistant GBM cells. Furthermore, Chk2, Nbs1 and MDC1 co-localized with γ-H2AX (data not shown), suggesting that these DDR proteins were intimately associated with one another in response to TMZ.

**Fig. 6:**
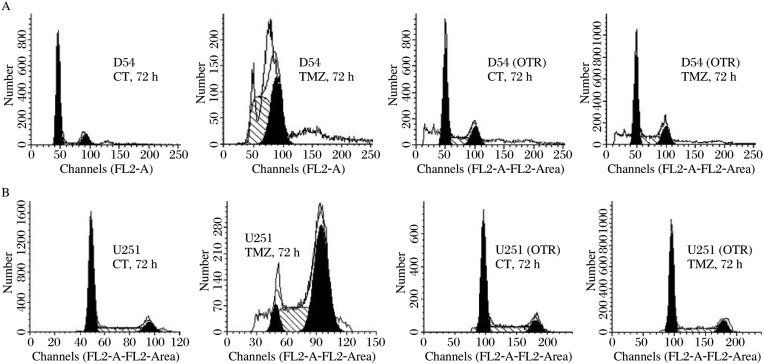
The resistant GBM cells are defective in cell cycle checkpoints. A: D54 (OTR) displays defective cell cycle checkpoints. B: U251 (OTR) exhibits defects in cell cycle checkpoints. These cells were harvested and processed for flow cytometric analysis of DNA content at 72 h post treatment. The cell cycle profiles shown in A and B are representative of at least three independent experiments. Solid areas, G1 or G2/M phase; diagonal lines, S phase. CT: control; TMZ: temozolomide.

### The resistant GBM cells showed defective cell cycle checkpoints

We investigated if impaired DDR signaling through the ATM-Chk2 pathway in the resistant GBM cells was associated with any defect in the activation of cell cycle checkpoints. Flow cytometric analysis revealed a marked rise in the percentage of U251 and D54 in the S phase and G2 phase 72 h post treatment (***Fig. 6A*** and ***6B***). The resistant GBM cells, on the other hand, exhibited virtually no change in cell cycle distributions compared with controls. These findings suggested that DNA lesions in the resistant GBM cells failed to trigger the activation of cell cycle checkpoints that was normally seen in cellular response to TMZ.

## DISCUSSION

TMZ has become part of standard care in combination with surgery and radiotherapy for GBM patients. However, resistance emerges with the prolonged use of the drug and the tumor often recurs in a more aggressive nature. Moreover, a significant subset of GBM patients shows clinical resistance to TMZ with operant mechanisms other than high MGMT activities or MMR deficiency.

TMZ causes alkylation damages and the DDR, which these lesions initiate, likely determines the fate of a tumor cell in survival or death. DNA alkylation damages inhibit DNA synthesis in the second S phase following treatment[26],[27]. We show that TMZ exhibits a biphasic inhibition of DNA synthesis: an initial acute but temporary inhibition followed by a more prolonged inhibition of DNA synthesis in the second S phase. In the acute phase, D54 and U251 exhibit a profile of inhibition of DNA synthesis similar to their resistant counterparts. In the prolonged phase, however, TMZ causes a greater depression of DNA synthesis in D54 and U251 compared with their TMZ-resistant counterparts, which exhibit MDS.

Though the decrease in the rate of DNA synthesis is small in this second phase, given the sharp increase in the proportion of S-phase cells in TMZ-treated D54 and U251, the degree of inhibition of DNA synthesis in these cells may be significantly greater than appreciated. Earlier studies indicated that this second S-phase inhibition of DNA replication was related to cytotoxicity of *O^6^*-meG adducts[17],[29]. In a GBM patient who suffered relapses, the recurrent tumors were less aggressive and highly sensitive to TMZ[30]. Intriguingly, TMZ caused a greater inhibition of DNA synthesis in the recurrent tumor samples than the primary tumor sample. These insights indicate that, in response to DNA alkylation damages, the GBM cells slow down ongoing DNA synthesis and allow the processing and repair of these DNA lesions, which, if unsuccessful, leads to the onset of cellular death. The lethality of this type of replication-arresting DMA damages may be due to futile, reiterative processing of *O^6^*-meG:C and *O^6^*-meG:T mispairs or due to attempted repair of DNA DSBs generated as a result of processing or repairing the primary DNA lesions[31]. This futile reiterative process leads to extensive lethalities of treated cells, and the more marked inhibition of DNA synthesis in D54 and U251 may, therefore, explain the greater sensitivity to TMZ in these cells than their resistant counterparts.

DNA lesions that are quickly repaired do not trigger the global DDR. However, we have observed similar extents of DNA alkylation damages between U251 and U251 (OTR) and between D54 and D54 (OTR), suggesting that DNA alkylation damages in the resistant GBM cells fail to signal to slow down ongoing DNA synthesis or that processing or repair of these DNA lesions is decoupled from DNA replication. *O^6^*-meG:T mismatches can only arise during the S phase. Alkylated DNA bases are incapable of pairing with any other natural bases, resulting in gaps or breaks on the newly synthesized DNA strand. TMZ also induces formation of DNA DSBs, which precede *O^6^*-meG-induced apoptosis in glioma cells, and replication is required for *O^6^*-meG-induced apoptosis[14],[16]. Though the resistant GBM cells display extensive alkylation damages, the failure to slow down ongoing DNA synthesis may be due to a failure to convert these DNA adducts into DNA strand breaks. However, this appears not to be the case as there exist similar amounts of DNA DSBs in U251 and U251 (OTR). DNA DSBs are also present in D54 (OTR) though to a smaller extent than those in D54. The presence of ongoing DNA alkylation damages and DNA strand breaks in the resistant GBM cells and their failure to trigger an inhibition of ongoing DNA synthesis unmask the presence of MDS in these cells.

Cancer-prone patients suffering from ataxia-telangiectasia exhibit radioresistant DNA synthesis (RDS)[32] and disruption of the ATM-Chk2 pathway results in defective S phase checkpoint and RDS[33]. The MRN complex is required for the activation of ATM and also functions downstream of ATM[34] and its deficiency was associated with defective Chk2 signaling in response to camptothecin-induced DNA DSBs[35]. The complex is also implicated in DDR to TMZ[19]. The DNA damage mediator protein MDC1 binds directly to Nbs1 and targets the latter to sites of DNA DSBs[20]. We show here that ATM, Chk2, MDC1, and Nbs1 form distinct foci in response to TMZ in U251 and D54 and these proteins also co-localize with γ-H2AX, suggesting that these DDR proteins are intimately associated with one another in response to TMZ. Though DNA alkylation damages and DSBs are present in the resistant GBM cells, γ-H2AX fails to localize to the sites of these DNA lesions. Additionally, these DNA damages fail to signal through the ATM-Chk2 pathway. MDC1 and Nbs1 also fail to form discrete foci in the resistant GBM cells.

Chk2 is implicated in irradiation-induced apoptosis[36],[37] and MDC1 interacts with Chk2 and is critical for Chk2-mediated apoptosis[38]. Chk2 also activates E2F-1 target genes which may determine the balance between cell survival and death[39],[40]. Furthermore, the suppression of TMZ-induced activation of Chk2 due to Akt overexpression protects GBM cells from TMZ-induced cytotoxicity[41]. Nbs1 is required for the induction of apoptosis by the MRN complex[42]. It also regulates apoptosis by disrupting the Ku70-Bax complex, leading to the activation of Bax and caspase-3[43]. The MRN complex has also been shown to contribute to TMZ-induced cytotoxicity[19]. MDS and the failed signaling through the ATM-Chk2 pathway and the lack of involvement of the MRN complex in DDR to TMZ may all contribute to the increased clonogenic survival that we have observed in the TMZ-resistant GBM cells.

Cell cycle checkpoints monitor the state of DNA and arrest cell cycle progression if DNA damage is detected and until a DNA lesion is repaired. If repair cannot be effected, the cell is committed to die. ATM, Chk2 and Nbs1 control the intra-S phase checkpoint in response to irradiation-induced DNA DSBs[44]. MDC1 interacts with Nbs1, which is required for irradiation-induced intra-S phase checkpoint[20]. The defective signaling through the ATM-Chk2 pathway and the failure of MDC1 and Nbs1 to form discrete foci in response to TMZ in the resistant GBM cells suggest that these cells exhibit defective cell cycle checkpoints. Glioma cells exhibited a robust cell cycle arrest in the S phase following treatment with 100 µmol/L TMZ[45]. We also observed a vigorous activation of an intra-S phase checkpoint following TMZ exposure in D54 and U251. However, we failed to observe any noticeable cell cycle distribution alterations in response to TMZ in the resistant GBM cells. These findings suggest that processing or repair of DNA alkylation damages and strand breaks is decoupled from cell cycle checkpoints or that these DNA lesions have escaped from the monitoring of cell cycle checkpoints.

Signaling of *O^6^*-meG lesions through the ATR-Chk1 pathway depends upon a functional MMR apparatus[12]. Our immunoblotting analysis indicates that the ATR-Chk1 is activated in all the GBM cell lines, suggesting that a functional MMR apparatus in these cells. We further showed that these GBM cells exhibited similar MMR capacity. The failed DDR signaling in the resistant GBM cells is, therefore, unlikely due to defective MMR. On the other hand, the failed DDR signaling through the ATM-Chk2 pathway in the resistant GBM cells may arise from a disconnection between the MMR complex and the MRN complex during DDR to TMZ. MMR has been implicated in S-phase checkpoint activation following ionizing irradiation and the MMR protein Msh2 was found to interact with Chk2 and Mlh1 was found to interact with ATM[46]. Additionally, Msh2 was required for the relocalization of Mre11 following irradiation and Msh2 defect led to defective Chk2 phosphorylation[47]. The MRN complex was also found to interact with Mlh1 in response to TMZ[19]. As the MRN complex is required for the activation of ATM[34], failure of the MMR apparatus to interact with the MRN complex may lead to defective phosphorylation by ATM of downstream effectors such as Chk2 or H2AX. There is also another distinct possibility that processing and repair of DNA lesions in the resistant GBM cells are decoupled from DDR to avoid triggering the onset of cellular death programs. The defective DDR signaling through the ATM-Chk2 pathway may also be due to a defect in an upstream regulator of ATM such as FOXO3a[48]. Silencing FOXO3a by siRNA was shown to impair ATM-regulated DDR signaling.

In conclusion, we have generated TMZ-resistant GBM cell lines whose resistance cannot be explained by high MGMT activities or MMR deficiency, the currently two known determinants of clinical response to TMZ in patients with GBM. Additionally, we have unmasked the presence of MDS in the TMZ resistant GBM cells. Furthermore, these resistant cells exhibit defective cell cycle checkpoints and impaired DDR signaling through the ATM-Chk2 pathway. As the functional availability of the DDR signaling network that responds to DNA damage likely directs how GBM responds to TMZ and decides the therapeutic outcome, the defective DDR signaling that we have observed likely drives the resistance in the temzolomide-resistant GBM cells.
